# Extracellular vesicles from *Lactobacillus druckerii* inhibit hypertrophic scar fibrosis

**DOI:** 10.1186/s12951-023-01861-y

**Published:** 2023-03-28

**Authors:** Fu Han, Kejia Wang, Kuo Shen, Jing Wang, Shichao Han, Dahai Hu, Gaofeng Wu

**Affiliations:** 1 Department of Burns and Cutaneous Surgery, Xijing Hospital, Air Force Medical University, Xi’an, 710032 Shaanxi China; 2 Department of Nursing, Air Force Medical University, Xi’an, 710032 Shaanxi China; 3 Department of Urology, Xijing Hospital, Air Force Medical University, Xi’an, 710032 Shaanxi China

**Keywords:** Hypertrophic scar, Extracellular vesicles, *Lactobacillus druckerii*, Probiotic, Fibrosis

## Abstract

**Background:**

Hypertrophic scars (HS) affect millions of people each year and require better treatment strategies. Bacterial extracellular vesicles (EVs) are advantaged by low cost and high yield which was commonly used in the treatment of diseases. Here, we investigated the therapeutic efficacy of EVs obtained from *Lactobacillus druckerii* in hypertrophic scar. In vitro, the effects of *Lactobacillus druckerii*-derived EVs (LDEVs) on Collagen I/III and α-SMA in fibroblasts obtained from HS. In vivo*,* a scleroderma mouse model was used to investigate the effects of LDEVs on fibrosis. The impact of LDEVs on excisional wound healing was explored. The different proteins between PBS and LDEVs treated fibroblasts derived from hypertrophic scar were studied by untargeted proteomic analysis.

**Results:**

In vitro, LDEVs treatment significantly inhibited the expression of Collagen I/III and α-SMA and cell proliferation of fibroblasts derived from HS. In vivo, LDEVs withdrawn the hypertrophic scar formation in scleroderma mouse model and decreased the expression of α-SMA. LDEVs promoted the proliferation of skin cells, new blood vessel formation and wound healing in excisional wound healing mice model. Moreover, proteomics has shown that LDEVs inhibit hypertrophic scar fibrosis through multiple pathways.

**Conclusions:**

Our results indicated that *Lactobacillus druckerii*-derived EVs has the potential application in the treatment of hypertrophic scars and any other fibrosis diseases.

**Graphical Abstract:**

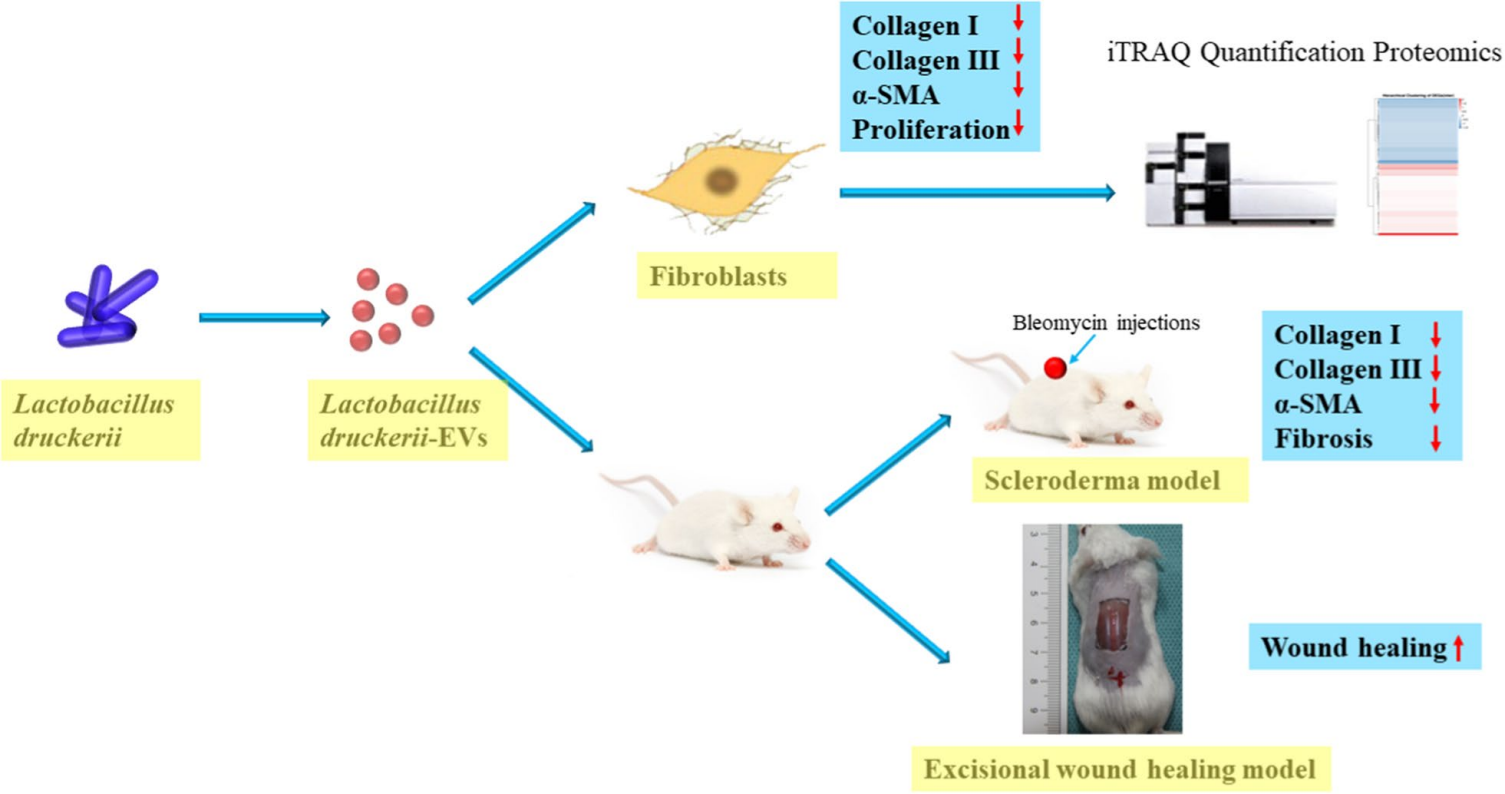

**Supplementary Information:**

The online version contains supplementary material available at 10.1186/s12951-023-01861-y.

## Introduction

Hypertrophic scar is one of the most common complications in burn patients, with an estimated incidence of up to 70% [1]. More than one million people worldwide are left with scars after surgery or trauma. Of these, 15% of scars are excessive and very challenging to treat [2]. There are a number of strategies for scar treatment. Such as, surgical excision, pressure garment therapy, bleomycin, fluorouracil, intralesional corticosteroid injections, Photodynamic therapy, Cryotherapy, Laser [3–6]. Treatment methods have certain side effects, such as skin atrophy, telangiectasia, pigmentation and skin ulcers. The disadvantage of silicone membrane therapy and pressure therapy is the long treatment time. The recurrence rate of surgical treatment is high. Laser therapy is not deep enough. Radiation therapy can lead to local skin color changes, systemic decolorization and other shortcomings [7]. Although there are many treatments for hyperplastic scars while is difficult to achieve achieving satisfactory results [8].

Extracellular vesicles (EVs) are small structures made of lipid membranes. It often envelop biomolecules released by cells produced in an environment composed of exosomes and microvesicles [9]. Various EVs derived from eukaryocyte, such as adipose-derived stem cell (ADSCs)-secreted EVs [10], adipose mesenchymal stem cell-secreted EVs [11], human-induced pluripotent stem cell-derived EVs [12], can inhibit the fibrosis of HS. Zhang et al. found that ADSCs-Exos can inhibit scar index [13]. uMSCs-Exos can promote wound regeneration, reduce skin fibrosis and scar formation [14]. However, many limitations of eukaryotic extracellular vesicles affect their clinical application, such as, large quantities of stem cell-conditioned medium on exosome production are limited [15], exosome production relies on animal serum to optimize cell growth [16], the high cost of mammal-derived cell culture, and medical ethics issues. Therefore, if EVs has the advantage of being fast, low cost, and available in large quantities which could made it has a more obvious advantage in clinical applications. Bacterial EVs are natural messengers involved in communication between microbial populations and cells within the species in microbiota [17]. Bacteria-derived EVs also can carry small RNAs, mRNAs, and proteins [18]. Bacteria-derived EVs can affect multifarious biological processes [19]. Bacteria-derived EVs are acknowledged to be proximally or distantly related to many human diseases [20–22]. The role of bacteria-secreted EVs in disease treatment has attracted the attention of researchers.

At present, many bacteria-derived EVs are used for the treatment of diseases. EVs secreted by *Escherichia coli* Nissle 1917 and ECOR63 can oppose the dysfunction of intestinal epithelial barrier [23]. EVs derived from *Lactobacillus plantarum*-derived can against ischemic brain injury [24]. *Lactobacillus reuteri* extracts promote wound healing [25]. EVs secreted from *Synechococcus elongatus* PCC7942 can accelerate cutaneous wound healing [26]. However, extracellular vesicles derived from probiotics have been rarely reported in scar treatment.

Hence, the current study was employed to determine whether EVs from *Lactobacillus druckerii* inhibit the fibrosis of hypertrophic scar. The influence of* L. druckerii* EVs (LDEVs) on hypertrophic scar fibrosis and related index was investigated by co-cultured with HS derived fibroblasts (HFBs) in vitro and used it treat scleroderma mouse model in vivo. Our results revealed that LDEVs could decrease the expression of Collagen I/III and α-SMA in vivo and in vitro. Additionally, LDEVs promote the proliferation of normal skin fibroblasts (NFBs) and inhibit the proliferation of HFBs. Moreover, LDEVs could enhance wound healing in excisional wound healing mouse model and reduce hypertrophic scar formation in scleroderma mouse model. Our findings suggest a potential therapeutic strategy to inhibit fibrosis in the clinical practices.

## Results

### Characterization of LDEVs

**Fig. 1:**
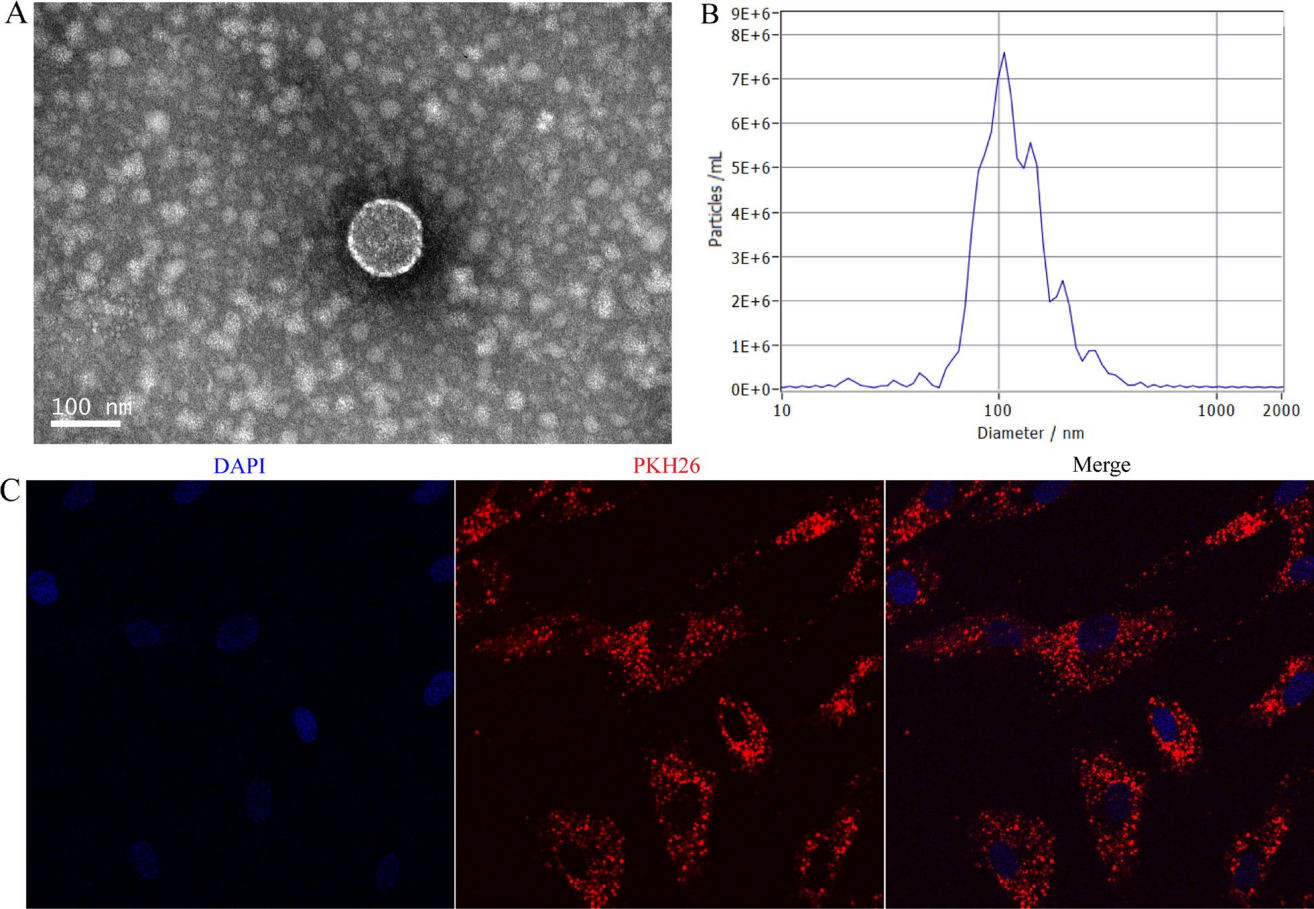
Characterization and uptake of LDEVs by HFBs. **A** Transmission electron microscopy imaging of LDEVs (scale bar = 100 nm). **B** Nanoparticle tracking analysis of LDEVs. **C** Representative images of the internalization of PKH-26-labeled LDEVs internalized by HFBs

LDEVs were isolated from the cell-free *L. druckerii* culture supernatants. TEM results indicated that LDEVs was the typical spherical vesicles morphology (Fig. 1A and Additional file 1: Fig. S1). NTA was used to analyze the diameter and concentration of LDEVs. The size of LDEVs ranged from approximately 80–130 nm in diameter (Fig. 1B and Additional file 1: Fig. S2). LDEVs were labeled with PHK26 and co-cultured with HFBs to determine whether LDEVs could be taken up by HFBs. As shown in Fig. 1C, PKH-26-labled LDEVs were traced in the perinuclear and nuclear region of HFBs. The results shown that PHK26-LDEVs were taken up by HFBs.

### LDEVs down-regulated the expression of fibrosis related-molecules

**Fig. 2:**
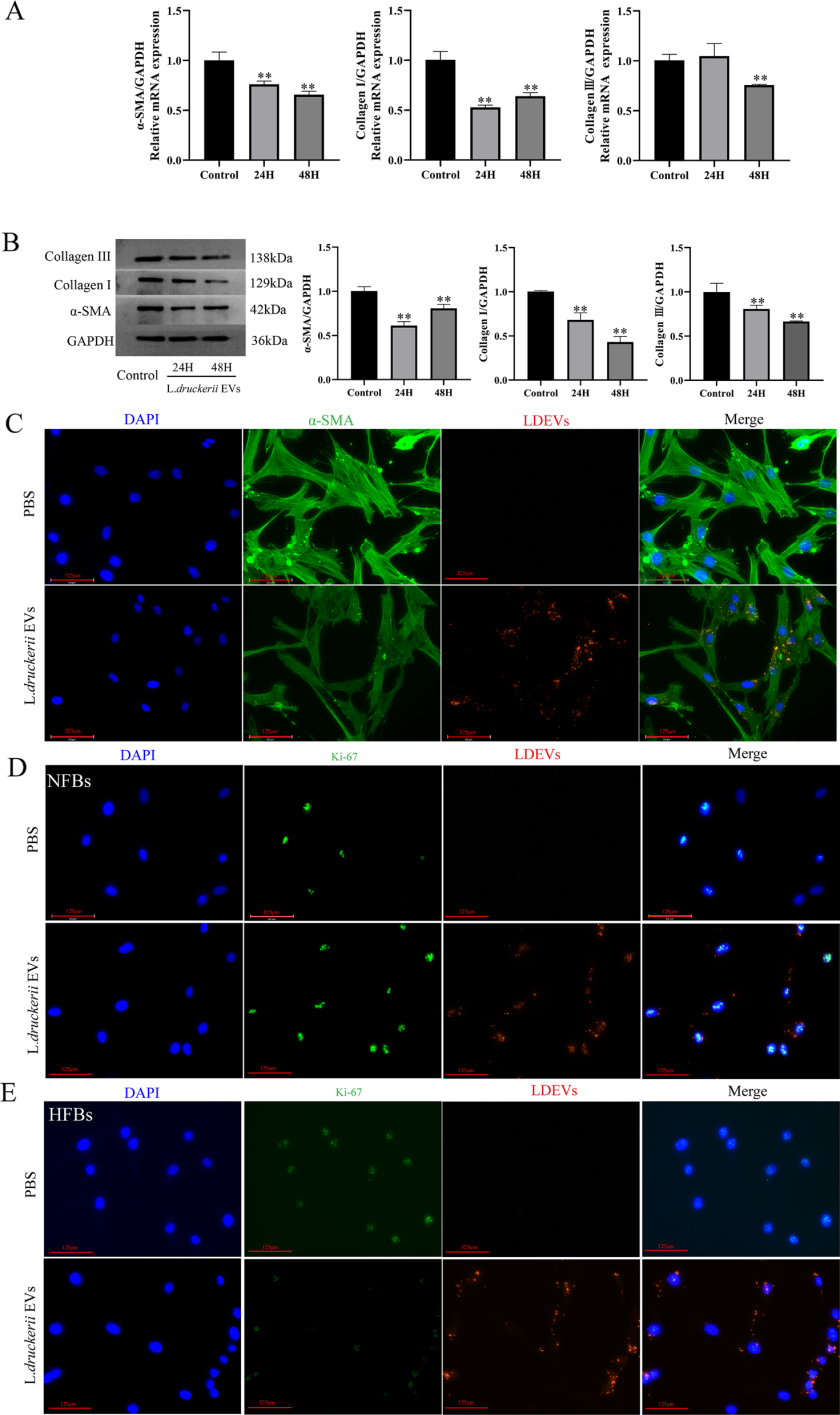
Effects of LDEVs on fibrosis of human hypertrophic scar fibroblasts. **A** qRT-PCR analysis of the fibrosis related factors in HFBs treated with LDEVs. **B** WB analysis of the fibrosis related factors in HFBs treated with LDEVs. **C** Representative images of α-SMA immunofluorescence staining in HFBs stimulated with LDEVs. scale bar = 125 μm. **D** Representative images of immunofluorescence staining of Ki-67 in HFBs and NFBs exposure to LDEVs or PBS, scale bar = 125 μm

HFBs were treated with LDEVs to investigate the influence of it on fibrosis. LDEVs treatment downregulate the expression of α-SMA and Collagen I/III both in mRNA (Fig. 2A) and protein level (Fig. 2B). The expression of α-SMA in HFBs was also investigated through immunofluorescence analysis, showing decreased levels of α-SMA expression after LDEVs treatment (Fig. 2C and Additional file 1: Fig. S3). Subsequently, we explored the effect of LDEVs on the proliferation of NFBs and HFBs through investigating the expression of Ki-67 by immunofluorescence. After treatment of NFBs with LDEVs, the expression Ki-67 was increased, while it was decreased in HFBs (Fig. 2D and Additional file 1: Fig. S4). The number of Ki-67-positive cells in LDEVs-treated group was enhanced than compared with PBS-treated (Additional file 1: Fig. S5). CCK8 and transwell assay results also shown that LDEVs inhibit the proliferation of HFBs (Additional file 1: Fig. S6A, B). We also investigated the influence of LDEVs on the expression collagen and α-SMA on NFBs. Results shown LDEVs didn’t affect the expression collagen I/III and α-SMA on NFBs (Additional file 1: Fig. S7). All results indicated that LDEVs could promote NFBs proliferation and inhibit HFBs. LDEVs was co-culture with RAW264.7 cells to investigate whether it can cause inflammation. Results indicated that LDEVs didn’t affect the expression of inflammatory factors (IL-1β, TNF-α, and IL-6) in RAW 264.7 cells (Additional file 1: Fig. S8). In conclusion, these data indicate that LDEVs could suppress HFBs differentiation and decrease the expression of fibrosis related index.

### LDEVs promotes the wound healing

**Fig. 3:**
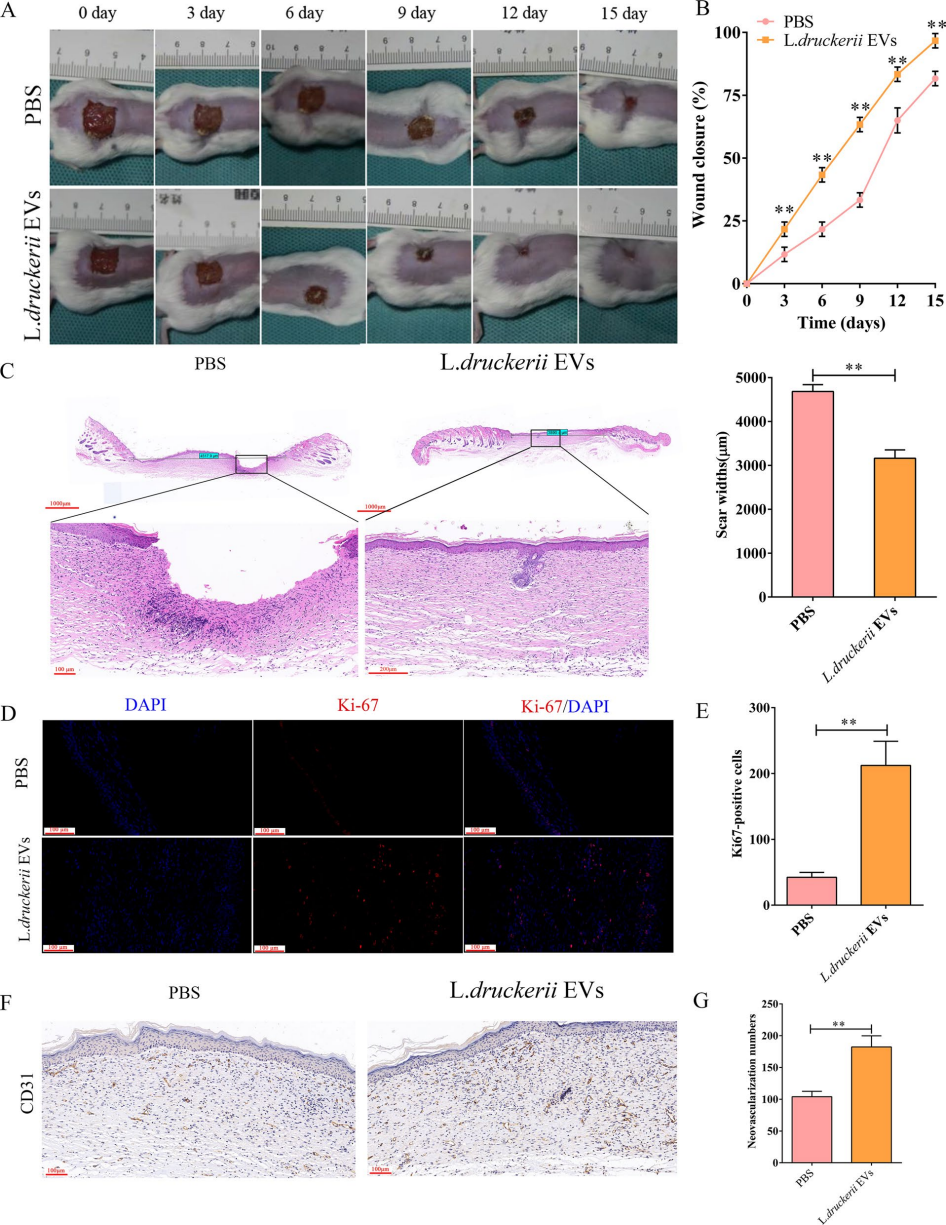
LDEVs accelerate the healing of cutaneous burn wounds. Representative images (**A**) and closure rate (**B**) of wounds treated with PBS, LDEVs at days 0, 3, 6, 9, 12 and 15 post-wounding. n = 10 per group. **C** Representative images of H&E-stained wound sections at day 15 post-wounding and quantification of the rate of re-epithelialization and scar widths. Representative images (**D**) and quantification (**E**) of skin cell proliferation by Ki-67 immunofluorescence staining. Scale bar: 100 μm. **F** Representative images of immunohistochemistry staining for CD31 and quantification (**G**) of neovascularization numbers. Data are plotted as mean ± SD. *P < 0.05, **P < 0.01

The speed of wound healing affects scar formation. Therefore, a full-thickness burn wounds mice model was established to investigate whether LDEVs can provide therapeutic benefits to cutaneous wounds. Representative digital photographs showed faster wound healing in LDEVs-treated mice compared to PBS-treated mice. The wound area was smaller in LDEVs-treated mice measured at days 3, 6, 9, 12 and 15 post-injury (Fig. 3A, B and Additional file 1: Fig. S9). H&E staining results showed that new epidermis and dermis, as well as regenerated hair follicles and adipocytes, took much longer to form in wounds injected with LDEVs than in PBS-treated controls on postoperative 15 day (Fig. 3C). The proliferation of skin cells in the wound sites was determined by Ki-67 immunostaining. It was shown that a great number of Ki-67-positive cells in LDEVs-treated wounds compared with PBS-treated wounds (Fig. 3D, E). CD31 is often used to evaluate the formation of the blood vessels. Therefore, we investigated the expression of CD31 in wound tissue of two groups of mice 15 days after modeling through CD31 immunohistochemical staining. The number of new capillaries in LDEVs group was meaningfully higher than PBS group (Fig. 3F, G). These results indicate that LDEVs is able to promote the healing of burn wounds.

### LDEVs attenuates scar formation in the mice HS model

**Fig. 4:**
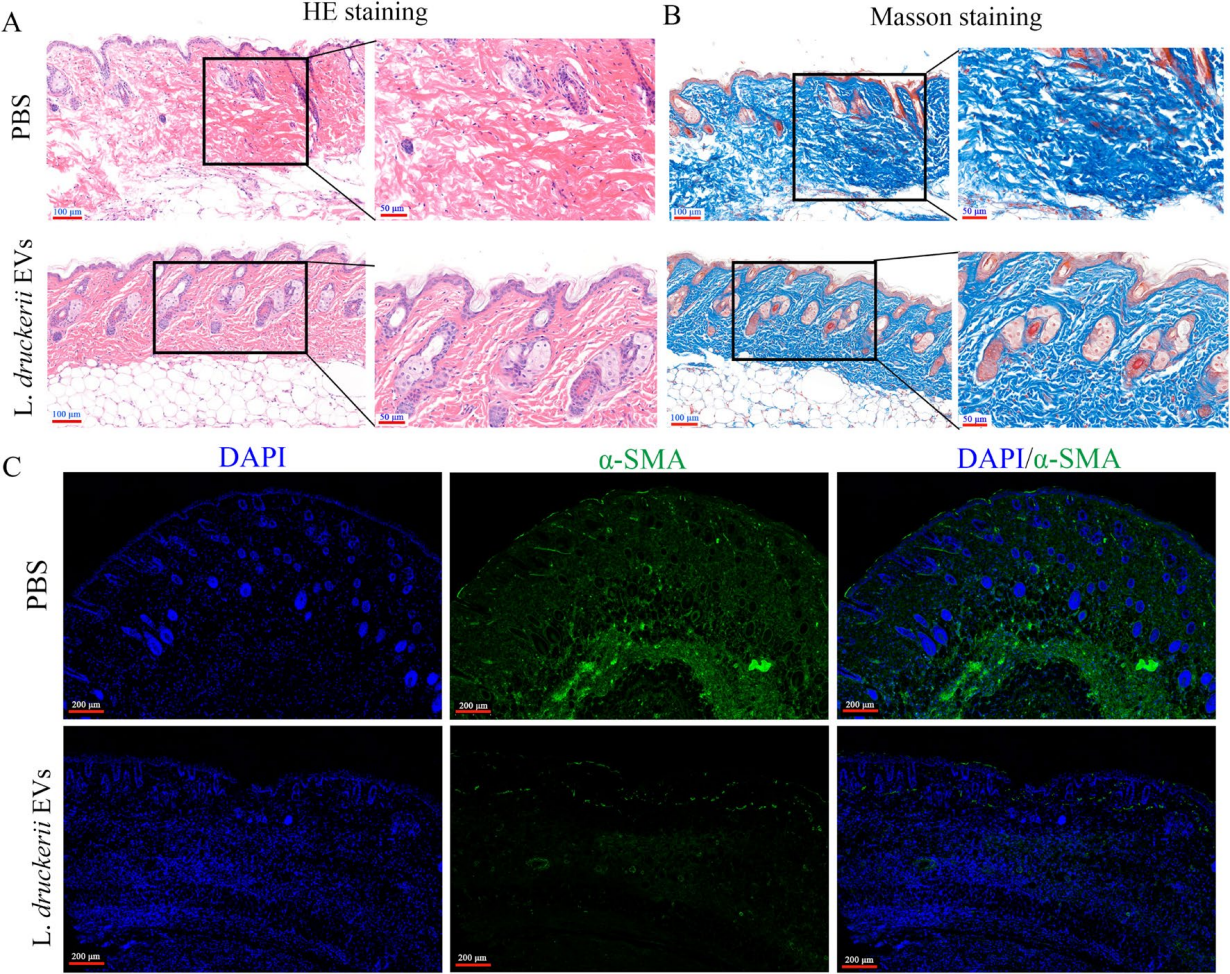
LDEVs attenuates excessive scarring in the mouse HS model (scleroderma mouse model). **A** HE staining showed that the hypertrophic scar features such as dermal thickening, hyperplastic epidermis, increased cellularity and reduction in the number of dermal appendages were significantly reversed after LDEVs treatment; scale bar: 100 μm and 50 μm. **B** Collagen deposition was attenuated in the LDEVs‐treated mice scars as shown by Masson staining; scale bar: 100 μm and 50 μm. **C** Representative images of immunofluorescence staining of α-SMA in skin tissues from scleroderma mouse model exposure to LDEVs or PBS, scale bar: 200 μm

We constructed scleroderma mouse model which was a common animal models of skin fibrosis and studied the effect of LDEVs on it. HE staining results found that LDEVs treatment reduced the thickness of dermis and epidermal hyperplasia. Moreover, LDEVs treatment reversed the increased cellularity and reduction in the number of dermal appendages (Fig. 4A). Scar collagen deposition in mice was also attenuated after LDEVs treatment (Fig. 4B). The expression of α-SMA decreased after LDEVs treatment through immunofluorescence analysis (Fig. 4C). These results indicated that LDEVs can attenuate hypertrophic scarring in vivo.

### Key differentially expressed proteins (DEPs) related to LDEVs inhibit fibrosis in HF

**Fig. 5:**
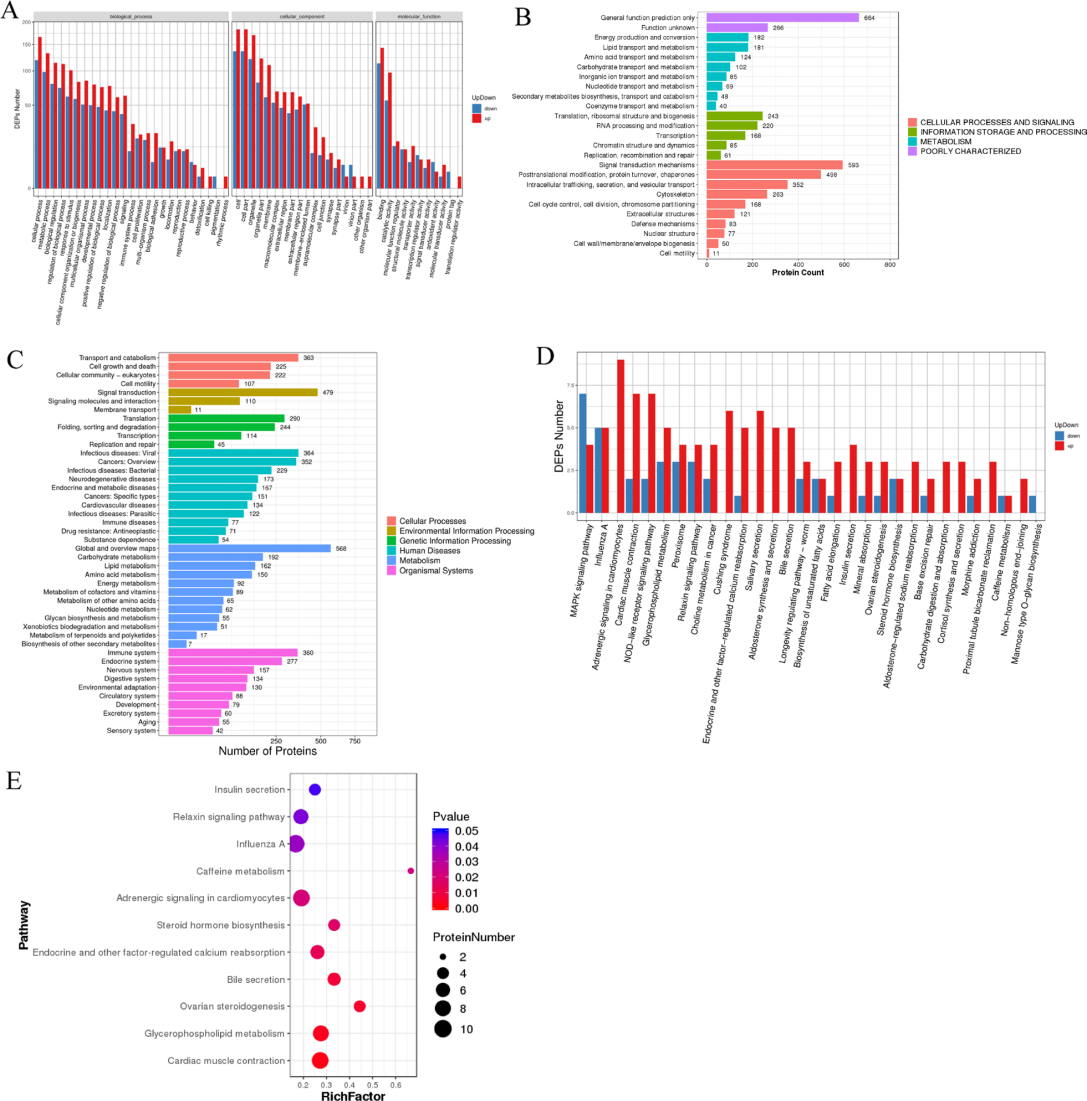
Inhibition fibrosis of LDEVs on HS at the proteome level. **A** The up and down statistical graph of GO Functional classification of differential proteins. x axis represents the GO annotation entries, y axis represents the number of up and down-regulated differential proteins. **B** KOG annotated differential proteins, y axis is the KOG entry, and the x axis is the number of proteins annotated by the corresponding KOG entry. **C** KEGG pathway functional enrichment of DEPs. x-axis represents enrichment factor. y-axis represents pathway name. **D** The up and down statistical graph of differential proteins in Pathway classification. The x-axis represents pathway annotation entries, and the y-axis represents the up and down-regulated number of DEPs. **E** Enriched pathway of metabolism of DEPs. The color indicates the P-value (high: blue; low: red), the lower P-value indicates the more significant enrichment. Point size indicates DEPs number. The bigger dots refer to larger amount

In order to investigate the mechanism of inhibition of hypertrophic scar fibrosis by LDEVs, we analyzed the differential expression proteins in LDEVs and PBS-treated HFBs by untargeted proteomic analysis. Results indicated that a total of 15,005 peptides matching 3805 proteins (with an FDR less than 1%) were identified. The GO term of these different expressed proteins was classified between the LDEVs group and control group. The differential proteins are mainly involved cellular process, metabolic process, biological regulation, binding, catalytic activity, molecular function regulator (Fig. 5A). KOGs annotation analysis shown that differential proteins are mainly involved in cellular processes and signaling, information storage and processing and metabolism (Fig. 5B). KEEG pathway annotation analysis showed differential proteins was related to signal transduction, immune system, endocrine system, nervous system, carbohydrate metabolism, lipid metabolism and amino acid metabolism (Fig. 5C). Pathway enrichment analysis indicated that differential proteins were mainly annotated to MAPK signaling pathway, adrenergic signaling in cardiomyocytes, cardiac muscle contraction, NOD-like receptor signaling pathway, Cushing syndrome, salivary secretion and bile secretion (Fig. 5D). MAPK pathway is a key cell signaling pathway involved in regulating cellular growth and proliferation [27]. We investigate the influence of LDEVs on the expression of p-JNK and p-p38 on the HFBs and NFBs. Results shown that LDEVs inhibit the expression of p-JNK and p-p38 in HFBs while promote it in NFBs (Additional file 1: Fig. S10). Results indicated that LDEVs might be reduce HFBs proliferation but promote NFBs growth through activate the MAPK pathway. Figure 5E shown the differential protein significantly enriched pathway of metabolism which were mainly annotated to insulin secretion, Relaxin signaling pathway, adrenergic signaling in cardiomyocytes, glycerophospholipid metabolism, cardiac muscle contraction pathway.

## Discussions

Hypertrophic scar is a serious skin fibrosis disease. There are many treatment methods for HS, including surgical resection, pressure therapy, radiation therapy, laser therapy, drug therapy. However, there are some side effects for these treatments, such as skin atrophy, pigmentation and skin ulcers, long treatment time and high recurrence rate. Therefore, it is particularly important for patients with HS to understand the mechanism of it and found treatment for HS from this point of view. The abnormal differentiation of myofibroblasts is one of the mechanisms of HS formation. In the study of the pathogenesis and development of hypertrophic scar, fibroblasts have been studied most extensively. The density and activity of fibroblasts are crucial for scar tissue formation, and their abnormal proliferation, differentiation, activation and apoptosis are involved in all stages of hypertrophic scar formation. Therefore, it is particularly important to inhibit the over-activation of fibroblasts and reduce fibrosis in the treatment of hypertrophic scar.

EVs are natural particles produced by almost all types of cells during life activities. A large number of extracellular vesicles are currently available for the treatment of hypertrophic scars, for example, adMSCs-Exos, BMSCs-Exos, uMSCs-Exos, MenSCs-Exos [28–31]. However, due to limited cell sources, slow in vitro expansion, potential immune rejection and medical ethics, EVs generated by these cells are difficult to form standardized products and industrialization. Therefore, bacterial extracellular vesicles have attracted the attention of many researchers. Lan Chu found that EVs derived from *Lactobacillus plantarum* can treat the ischemic stroke [24]. EVs derived from *Lactobacillus reuteri* attenuated LPS-induced inflammation [32]. *Escherichia coli* strain Nissle 1917 OMVs could reduce intestinal inflammation [23]. In the present study, EVs were obtained from the supernatant of *Lactobacillus druckerii* culture through differential ultra-high speed centrifugal method. The LDEVs diameter was 80–130 nm, which was consistent with the size of bacterial extracellular vesicles reported in the previous studies [33, 34]. We found that LDEVs can reduce Collagen I/III and α-SMA expression in HFBs, promote wound healing and inhibit scar formation. The researchers found that outer membrane vesicles (OMVs) derived from *Helicobacter pylori*-derived have a probable role in liver fibrosis progression [35]. *Akkermansia muciniphila*-secreted EVs can inhibit the expression of liver fibrosis markers [36]. In the mouse model of excision wound healing, LDEVs can promote wound healing, and the speed of wound healing is one of the key factors affecting scar formation. Therefore, LDEVs holds great prospect for the treatment of fibrosis. Bacterial derived extracellular vesicles have the following advantages in treating diseases compared to eukaryotic or serum-derived extracellular vesicles. The rate of bacterial reproduction is fast, and it is easy to obtain and culture the supernatant in large quantities to produce extracellular vesicles on a large scale. Bacteria-EVs contain different classes of biomolecules such as nucleic acids, lipids, proteins, and diverse types of small molecular metabolites [37, 38]. Bacteria-EVs may be treat diseases through those biomolecules. In this study, we found that LDEVs can inhibit fibrosis while we did not study the specific components of LDEVs that inhibit fibrosis which can be studied in the future.

LDEVs may inhibit scar fibrosis by affecting a variety of pathways. Proteomics can reflect the changes of all proteins, providing a possible mechanism for EVs in disease treatment. In the current study, proteomics analysis was performed to observe the effects of LDEVs on HS, predicting the potential targets or relevant paths as a whole. The analysis indicated that the different proteins between LDEVs treatment group and control group were mainly enriched into MAPK signaling pathways, adrenergic signaling in cardiomyocytes, NOD-like receptor signaling pathway, Cushing syndrome, salivary secretion and bile secretion, insulin secretion, Relaxin signaling pathway, glycerophospholipid metabolism, cardiac muscle contraction pathway. These results suggest that they may be involved in the regulatory role of LDEVs in inhibiting HS fibrosis. Many literatures have reported that MAPK signaling pathway is related to fibrotic disease [39, 40]. MAPK pathway is also a key cell signaling pathway involved in regulating cellular growth and proliferation [27]. We found that LDEVs can reduce HFBs proliferation but promote NFBs growth through activate the MAPK pathway. Of course, LDEVs may affect HFBs and NFBs proliferation by affecting the expression of other signaling pathways. Lin et al. found that tormentic acid alleviated liver injury and fibrosis by regulating glycerophosphatide metabolic pathway, suggesting that it plays an important role in fibrotic diseases [41]. Previous studies have shown that defective insulin secretion in cystic fibrosis affects growth before hyperglycemia occurs, which is also confirmed by another study [42, 43]. Many researchers found that bile secretion was related to fibrosis disease [44]. Zhang et al. found that B7-33 which targets relaxin family peptide receptors can activate hepatic stellate cells (HSCs) can weaken the fibrogenic properties of activated HSCs [45]. Adrenergic signaling has been implicated in cancer initiation, progression [46]. Of course, the specific molecular mechanism of LDEVs inhibiting HS fibrosis was obtained through proteomic analysis. As for the specific mechanism, we will determine it by gene knockout and high-throughput sequencing technology. In addition, the specific components of LDEVs have not been studied in detail in this paper. In the future research work, exosome sequencing technology can be used to identify the specific components, so as to understand which components play the role of inhibiting fibrosis.

## Conclusion

In recent years, with the increasing number of patients with hypertrophic scar and some defects in the current conventional treatment methods, it is urgent to develop new treatment methods for HS. Extracellular vesicles (EVs) have attracted the interest of many researchers, but the clinical application value of EVs from eukaryotic cells is limited due to some problems such as ethics and difficulty in culture. Therefore, EVs from bacteria have attracted more attention. Based on our results, the EVs derived from *Lactobacillus druckerii* decreased the expression of Collagen I, Collagen III and α-SMA in HFBs isolated and cultured from HS patients. LDEVs also promote the wound healing in a murine model of excisional wound healing and inhibit scar formation in scleroderma mouse model. Proteomic results showed that LDEVs may inhibit HS fibrosis through a variety of pathways. Our findings provide a theoretical basis for the treatment of EVs derived from *Lactobacillus druckerii* in hypertrophic scars. Moreover, our study provides a new strategy for hypertrophic scars and other fibrosis disease treatment.

## Materials and methods

### Isolation and identification of LDEVs

*Lactobacillus druckerii* was cultured in MRS growth medium (Solarbio Science & Technology, Beijing, China) supplemented with 0.5% glucose. EVs were obtained from the *L. druckerii* culture supernatant. Briefly, *L. druckerii* was grown at 37 °C for 12 h until the OD 600 nm reach to 0.9–1.0 which the start OD 600 nm was 0.2. The cell culture was centrifugated for 20 min (12,000×*g*) at 4 °C. Large particles in the supernatant was removed by filtered through a 0.45-μm membrane. Then, the solution was filtered through a 0.22-μm membrane and centrifugation for 10 min (2000×*g*) at 4 °C. The supernatant was transferred to a new clean centrifuge tube and centrifuged 10 min at 4 °C (10,000×*g*). Then, the supernatant was moved to another centrifuge tube and ultracentrifuged for 75 min at 110,000×*g* at 4 °C. 1 mL sterile phosphate buffer saline was used to resuspend the precipitate and filtered through 0.22-μm membrane. Then the solutions were ultracentrifuged for 75 min (110,000×*g*) at 4 °C. 1 mL sterile phosphate buffer saline to resuspend the precipitate and store at − 80 °C. Nanoparticle tracking analysis (NTA) was used to determine the diameter size and particle number of LDEVs (ZetaVIEW S/N 17-310, PARTICLE METRIX). Morphological characteristics of LDEVs were detected with transmission electron microscopy (TEM) using Tecnai G2 Spirit BioTwin at 80 kV (FEI).

### Labeling of LDEVs and imaging observation

PKH26 kit was used to label the Fluorescent of LDEVs (Sigma, St. Louis, MO, USA) according to product description. PKH26-marked LDEVs were co-cultured with hypertrophic scar derived fibroblasts (HFBs) for 24 h. Then, HFBs were fixed and stained with 4′,6-diamidino-2-phenylindole (DAPI) and the stained images were observed under confocal microscopy.

### Cell culture and treatment

Human tissues were gained from 8 volunteer patients which average age was 20 years and ranging from 7 years old to 42 years. All patients were from Department of Burns and Cutaneous Surgery, Xijing Hospital, Air Force Medical University (Xi'an, China) who was underwent surgical excision. For each patient, the nature of the HS was established by three clinicians. HFBs were isolated according to previous methods [47]. NFBs were isolated from skin biopsy samples as previously described. HFBs and NFBs (P3–P6) were used for experimental studies. Cells were cultured in 6-well culture plates with 1 × 10^4^ cells per well. LDEVs was used to treat HFBs and NFBs for 24 h and 48 h. RAW264.7 cells were used to investigated the influence of LDEVs on the inflammation. RAW264.7 cells were cultured in 6-well culture plates with 1 × 10^6^ cells per well and used LDEVs to treat it for 24 h. The cells and cell supernatants were collected to analysis the expression of IL-1β, TNF-α and IL-6.

### CCK8 assays

The influence of LDEVS on HFBs proliferation was measured by the cell counting kit-8 (Beyotime Biotechnology, Shanghai, China) following the manufacturer’s instructions. 2000 HFBs per well were seeded in 96-well plates and cultured with PBS or LDEVs for 24 h and 48 h. Then CCK8 solution were added and absorbance at 450 nm were determined through a microtiter plate reader (Infinite 200 PRO, Switzerland).

### Mouse model and treatment

6–8 weeks old BALB/c mice weighing 22–25 g (n = 20) were used in this study. A murine model of excisional wound healing was chosen to investigate the influence of LDEVs on wound healing. In brief, the backs of mice were shaved and cleaned after the mice were anesthetized. A 1.5 cm^2^ full-thickness wound was shaped on the mouse dorsum. Animals (n = 10/group) were randomly divided into two groups and injected subcutaneously with LDEVs or PBS at four injection sites (25 μL each).

Scleroderma mouse model was made according to the protocol previous. Briefly, BALB/c mice (n = 24) were shaved (approximately 2.0 cm × 2.0 cm) from the same part of the central back. Each group was injected with bleomycin hydrochloride buffer (1 mg/mL), 0.1 mL once a day, for 28 consecutive days. Mice were randomly divided into three groups (n = 8): control group (PBS injected), LDEVs treat group (25 μL and 50 μL injected).

### qRT-PCR

Trizol was used to extract the Total RNA of tissues and cells (Takara, Japan). cDNA was obtained by PrimeScript™ RT reagent Kit (Takara, Japan). The 2^−ΔΔCT^ method was used to analyze the relative gene expression which GAPDH was used as internal controls. qRT-PCR was performed by qRT-PCR system (BioRad, Singapore). Primer sequences used for this study were as follows: *GAPDH*: forward, 5′-CACCATGGAGAAGGCCGGGG-3′, and reverse, 5′-GACGGACACATTGGGGG TAG-3′; *α-SMA*: forward, GACAATGGCTCTGGGCTCTGTAA, and reverse, TGT GCTTCGTCACCCACGTA; *Collagen I*: forward, GAGGGCAACAGCAGG TTCACTTA, and reverse, TCAGCACCACCGATGTCCA; *Collagen III*: forward, CCACGGAAACACTGGTGGAC, and reverse, GCCAGCTGCACATCAAGGAC. *IL-1β:* forward, GCTTCAGGCAGGCAGTATC, and reverse, AGGATGGGCTCTTCT TCAAAG; *TNF-α:* forward, AGAGCTACAAGAGGATCACCAGCAG, and reverse, TCAGATTTACGGGTCAACTTCACAT; *IL-6*: forward, GAGGATACCACTCCCAA CAGACC and reverse, AAGTGCATCATCGTTGTTCATACA.

### Western blot analysis

HFBs after different treatments were washed with ice-cold PBS three times. 80 μL RIPA buffer containing protease and phosphatase inhibitor was added to lyse the cells and centrifugated for 10 min (14,000×*g*) at 4 °C. 50 mg total protein determined by BCA was processed by SDS-PAGE and transferred to polyvinylidene difluoride (PVDF) membrane (Millipore, Bedford, MA, USA). Membranes were blocked with 5% non-fat milk and incubated with different antibodies anti-Collagen III (1:1000, Abcam, UK) anti-Collagen I (1:1000, Abcam, UK), anti-SMA (1:1000, CST, USA). The membranes were imagined with ECL detection system (Alpha Innotech, San Leandro, CA).

### Histological, immunofluorescent and immunohistochemistry analyses

Skin tissue samples were fixed in 10% formalin, dehydrated with ethanol and embedded in paraffin. Then, samples were cut into 4 μm-thick sections. Sections go through the following steps: deparaffinized in xylene, rehydrated through decreasing concentrations of ethanol and distilled water. At last, the sections were subjected to further analysis. Hematoxylin and eosin (H&E) and Masson’s trichrome were used for histological analysis.

Sections were incubated with autofluorescence quencher for 5 min after antigen extraction by boiling the sections in EDTA-Tris buffer (pH 9.0), then blocked with 3% BSA for 30 min at room temperature, followed by incubated with anti-Ki67 primary antibody (1:100; CST, USA) at 4 °C for 12 h. Sections was washed with PBS three times and then incubated with secondary antibody (1:250; Abcam). Nuclei were stained with DAPI (0.5 µg/mL; Invitrogen, Carlsbad, USA). The pictures were achieved by fluorescence microscope (FV1000, Olympus, Japan). Three random visual fields were used to examine the Ki67-postive cells of different sections.

Rehydrated sections which were used for immunohistochemistry (IHC) staining were heated in EDTA-Tris buffer (pH 9.0) for 15 min by microwave. Then samples were blocked with 3% hydrogen peroxide for 25 min. Then, the sections were blocked with 3% BSA for 30 min and incubated with the primary antibody anti-CD31 (1:50; CST) overnight at 4 °C. The immunoactivity was explored by horseradish peroxidase IHC detection system (Servicebio) and then counterstained with hematoxylin. Three random fields were used in each section to count the number of CD31-positive vessels.

### Protein preparation and iTRAQ labeling

HFBs with or without LDEVs treatment were washed with ice-cold PBS three times. Then cells were lysed with RIPA buffer containing with protease and phosphatase inhibitor and then centrifuged for 10 min (14,000×*g*) at 4 °C. Bradford method was used to determine the concentration of proteins. Protein samples were stored at − 80 °C until used. The method of proteomics was supplied by BGI (Shen Zhen, China). First, each sample was digested with Trypsin Gold (Promega, Madison, WI, USA) for 12 h at 37 °C. Second, samples were desalted and dried under vacuum conditions. Next, the resuspended peptides were marked with new iTRAQ Reagent 8-plex (AB Sciex, Foster City, CA, USA). The mixed peptides were separated on a Shimadzu LC-20AB HPLC Pump system, desalted and vacuum-dried. The peptides separated from nanoHPLC (Thermo Scientific™ UltiMate™ 3000 UHPLC system) were subjected into the tandem mass spectrometry QEXACTIVE HF X (Thermo Fisher Scientific, San Jose, CA) for DDA (data-dependent acquisition) detection by nano-electrospray ionization.

### Statistical analysis

The experiment was repeated at least three times, and each experiment was presented as mean ± SD. Student’s t test was used for statistical differences between paired samples, and one-way analysis of variance (ANOVA) was used for comparison between multiple groups. All analyses were performed using Prism 8 software (GraphPad). A P-value < 0.05 was considered as statistically significant.

## Supplementary Information

**Additional file 1: Figure S1.** Transmission electron microscopy imaging of LDEVs (scale bar = 200 nm). **Figure S2.** Nanoparticle tracking analysis of the sample from PBS. **Figure S3.** Representative images of α-SMA immunofluorescence staining in HFBs stimulated with LDEVs. scale bar = 500 μm. **Figure S4.** Representative images of immunofluorescence staining of Ki-67 in HFBs and NFBs exposure to LDEVs or PBS, scale bar = 125 μm. **Figure S5.** Quantification Ki-67 positive cells from NFBs and HFBs after PBS and LDEVs treatment. **Figure S6.** LDEVs inhibit HFBs proliferation according to CCK8 assay (A) and Transwell assay (B). **Figure S7.** qRT-PCR analysis of the fibrosis related factors (Collagen I, Collagen III and α-SMA) in HFBs treated with LDEVs. **Figure S8.** The expression of inflammatory factors (IL-1β, TNF-α, and IL-6) in RAW264.7 cells and the cell supernatants of RAW264.7 cells after LDEVs treatment. **Figure S9.** Representative images of wounds treated with PBS, LDEVs at days 0, 3, 6, 9, 12 and 15 post-wounding. **Figure S10.** The influence of LDEVs on the expression of MAPK signaling pathway in HFBs and NFBs.

## Data Availability

The datasets during and/or analyzed during the current study available from the corresponding author on reasonable request.
