# Supplementary material for: Extracellular vesicles from Lactobacillus druckerii inhibit hypertrophic scar fibrosis
Source: J Nanobiotechnology. 2023 Mar 28;21:113. doi: 10.1186/s12951-023-01861-y (PMC10053340; doi:10.1186/s12951-023-01861-y)
Supplement: Supplementary file 1 — Additional file 1: Figure S1. Transmission electron microscopy imaging of LDEVs (scale bar = 200 nm). Figure S2. Nanoparticle tracking analysis of the sample from PBS. Figure S3. Representative images of α-SMA immunofluorescence staining in HFBs stimulated with LDEVs. scale bar = 500 μm. Figure S4. Representative images of immunofluorescence staining of Ki-67 in HFBs and NFBs exposure to LDEVs or PBS, scale bar = 125 μm. Figure S5. Quantification Ki-67 positive cells from NFBs and HFBs after PBS and LDEVs treatment. Figure S6. LDEVs inhibit HFBs proliferation according to CCK8 assay (A) and Transwell assay (B). Figure S7. qRT-PCR analysis of the fibrosis related factors (Collagen I, Collagen III and α-SMA) in HFBs treated with LDEVs. Figure S8. The expression of inflammatory factors (IL-1β, TNF-α, and IL-6) in RAW264.7 cells and the cell supernatants of RAW264.7 cells after LDEVs treatment. Figure S9. Representative images of wounds treated with PBS, LDEVs at days 0, 3, 6, 9, 12 and 15 post-wounding. Figure S10. The influence of LDEVs on the expression of MAPK signaling pathway in HFBs and NFBs. [file 12951_2023_1861_MOESM1_ESM.docx]

**Additional file 1**


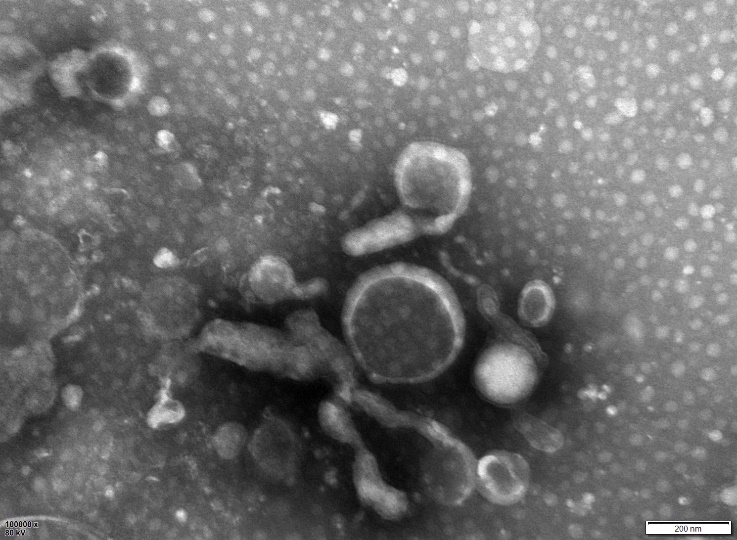


**Figure S1** Transmission electron microscopy imaging of LDEVs (scale bar = 200 nm).


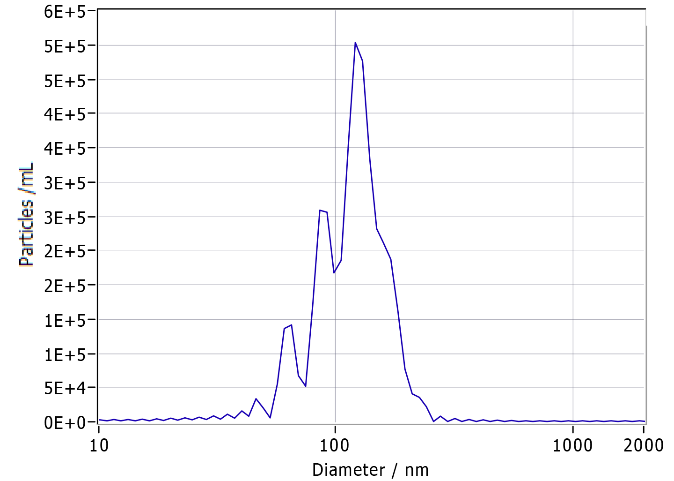


**Figure S2** Nanoparticle tracking analysis of the sample from PBS.


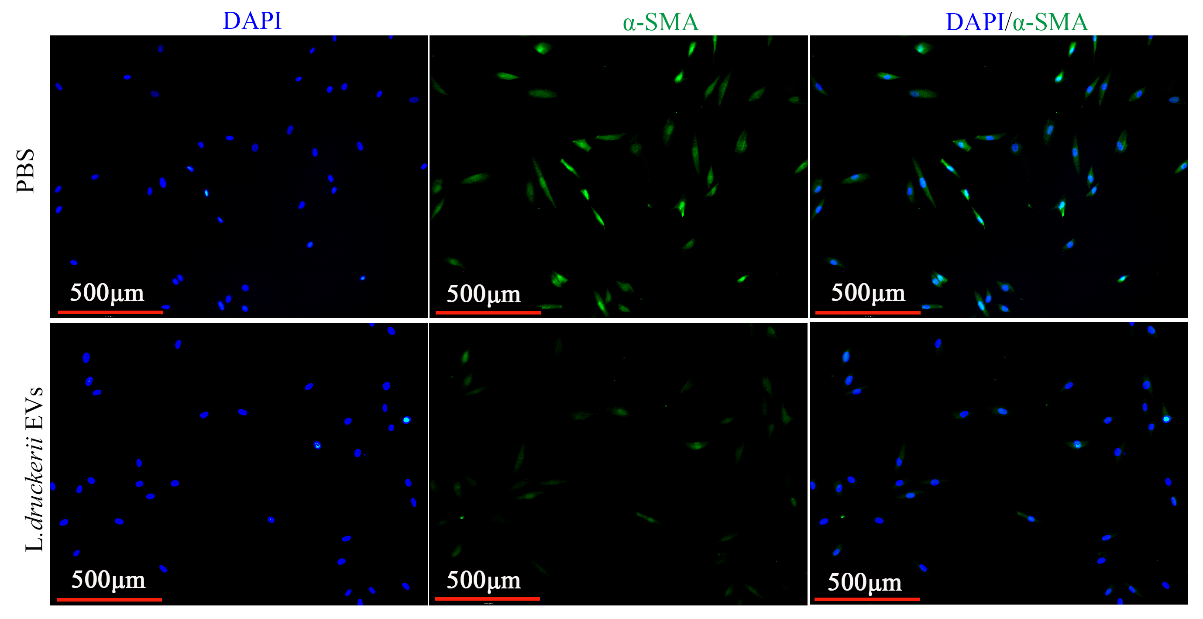


**Figure S3** Representative images of α-SMA immunofluorescence staining in HFBs stimulated with LDEVs. scale bar = 500 μm.


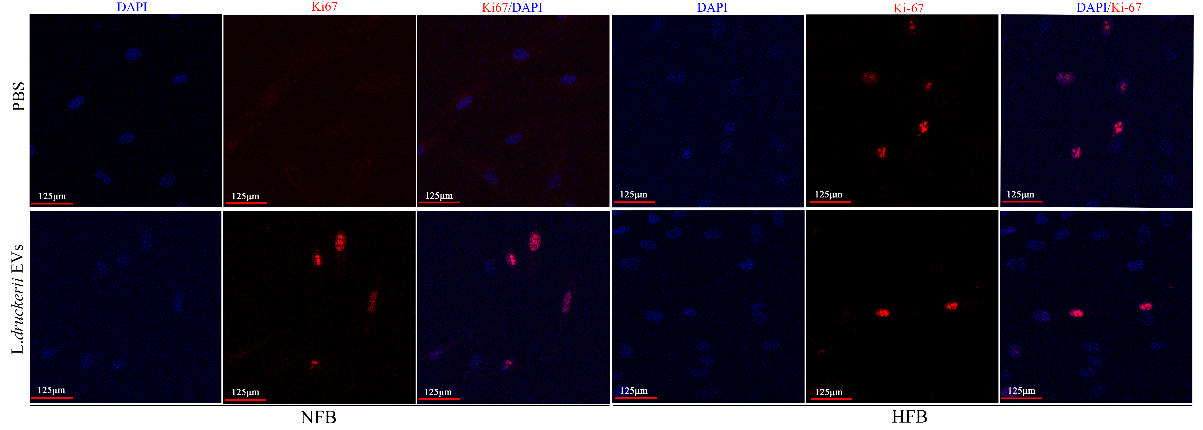


**Figure S4** Representative images of immunofluorescence staining of Ki-67 in HFBs and NFBs exposure to LDEVs or PBS, scale bar = 125 μm.





**Figure S5** Quantification Ki-67 positive cells from NFBs and HFBs after PBS and LDEVs treatment.


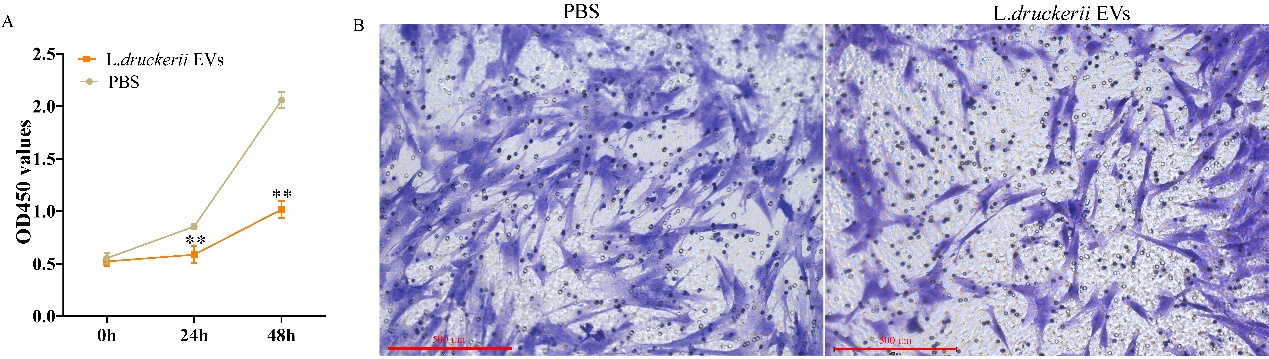


**Figure S6** LDEVs inhibit HFBs proliferation according to CCK8 assay (A) and Transwell assay (B).





**Figure S7** qRT-PCR analysis of the fibrosis related factors (Collagen I, Collagen III and α-SMA) in HFBs treated with LDEVs.





**Figure S8** The expression of inflammatory factors (IL-1β, TNF-α, and IL-6) in RAW264.7 cells and the cell supernatants of RAW264.7 cells after LDEVs treatment.


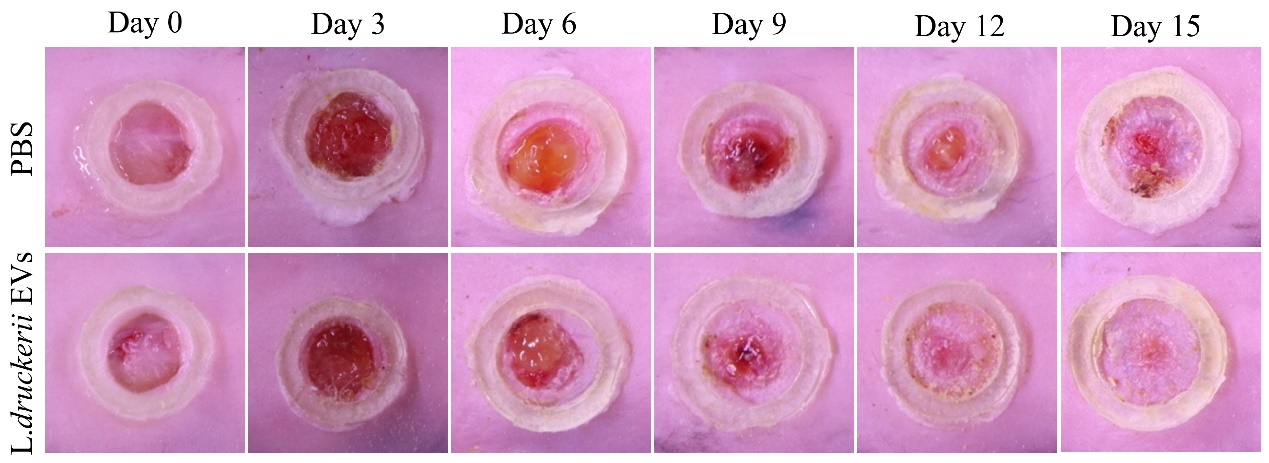


**Figure S9** Representative images of wounds treated with PBS, LDEVs at days 0, 3, 6, 9, 12 and 15 post-wounding.


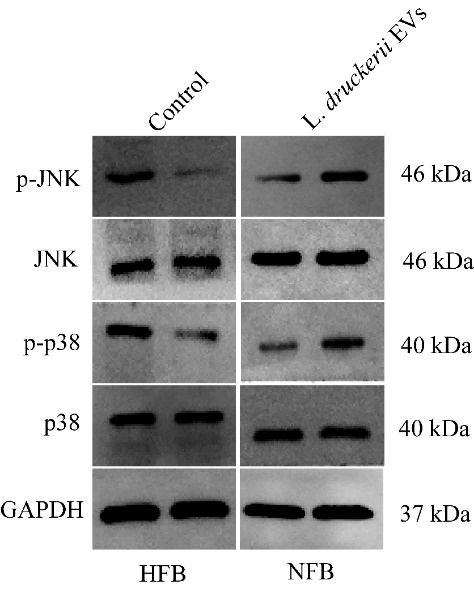


**Figure S10** The influence of LDEVs on the expression of MAPK signaling pathway in HFBs and NFBs.
